# Structural Characterization of CYP51 from *Trypanosoma cruzi* and *Trypanosoma brucei* Bound to the Antifungal Drugs Posaconazole and Fluconazole

**DOI:** 10.1371/journal.pntd.0000651

**Published:** 2010-04-06

**Authors:** Chiung-Kuang Chen, Siegfried S. F. Leung, Christophe Guilbert, Matthew P. Jacobson, James H. McKerrow, Larissa M. Podust

**Affiliations:** 1 Department of Pharmaceutical Chemistry, University of California, San Francisco, California, United States of America; 2 Sandler Center for Basic Research in Parasitic Diseases, University of California, San Francisco, California, United States of America; McGill University, Canada

## Abstract

**Background:**

Chagas Disease is the leading cause of heart failure in Latin America. Current drug therapy is limited by issues of both efficacy and severe side effects. *Trypansoma cruzi*, the protozoan agent of Chagas Disease, is closely related to two other major global pathogens, *Leishmania spp*., responsible for leishmaniasis, and *Trypansoma brucei*, the causative agent of African Sleeping Sickness. Both *T. cruzi* and *Leishmania* parasites have an essential requirement for ergosterol, and are thus vulnerable to inhibitors of sterol 14α-demethylase (CYP51), which catalyzes the conversion of lanosterol to ergosterol. Clinically employed anti-fungal azoles inhibit ergosterol biosynthesis in fungi, and specific azoles are also effective against both *Trypanosoma* and *Leishmania* parasites. However, modification of azoles to enhance efficacy and circumvent potential drug resistance has been problematic for both parasitic and fungal infections due to the lack of structural insights into drug binding.

**Methodology/Principal Findings:**

We have determined the crystal structures for CYP51 from *T. cruzi* (resolutions of 2.35 Å and 2.27 Å), and from the related pathogen *T. brucei* (resolutions of 2.7 Å and 2.6 Å), co-crystallized with the antifungal drugs fluconazole and posaconazole. Remarkably, both drugs adopt multiple conformations when binding the target. The fluconazole 2,4-difluorophenyl ring flips 180° depending on the H-bonding interactions with the BC-loop. The terminus of the long functional tail group of posaconazole is bound loosely in the mouth of the hydrophobic substrate binding tunnel, suggesting that the major contribution of the tail to drug efficacy is for pharmacokinetics rather than in interactions with the target.

**Conclusions/Significance:**

The structures provide new insights into binding of azoles to CYP51 and mechanisms of potential drug resistance. Our studies define in structural detail the CYP51 therapeutic target in *T. cruzi*, and offer a starting point for rationally designed anti-Chagasic drugs with improved efficacy and reduced toxicity.

## Author Summary

Chagas Disease is caused by kinetoplastid protozoa *Trypanosoma cruzi,* whose sterols resemble those of fungi, in both composition and biosynthetic pathway. Azole inhibitors of sterol 14α-demethylase (CYP51), such as fluconazole, itraconazole, voriconazole, and posaconazole, successfully treat fungal infections in humans. Efforts have been made to translate anti-fungal azoles into a second-use application for Chagas Disease. Ravuconazole and posaconazole have been recently proposed as candidates for clinical trials with Chagas Disease patients. However, the widespread use of posaconazole for long-term treatment of chronic infections may be limited by hepatic and renal toxicity, a requirement for simultaneous intake of a fatty meal or nutritional supplement to enhance absorption, and cost. To aid our search for structurally and synthetically simple CYP51 inhibitors, we have determined the crystal structures of the CYP51 targets in *T. cruzi* and *T. brucei*, both bound to the anti-fungal drugs fluconazole or posaconazole. The structures provide a basis for a design of new drugs targeting Chagas Disease, and also make it possible to model the active site characteristics of the highly homologous *Leishmania* CYP51. This work provides a foundation for rational synthesis of new therapeutic agents targeting the three kinetoplastid parasites.

## Introduction

Chagas Disease, a potentially lethal tropical infection, is caused by the kinetoplastid protozoan *Trypanosoma cruzi*, which is spread by blood-sucking reduviid insects [1]. It is the leading cause of heart failure in Latin America, with an estimated to 8–10 million people infected [2]. The parasite invades and reproduces in a variety of host cells, including macrophages, smooth and striated muscle, fibroblasts and neurons. Disease progression is marked by an initial acute phase, which typically occurs in children, followed by a symptom-free intermediate phase. A chronic phase leading to GI tract lesions and heart failure often ensues. Current chemotherapy options are limited to nifurtimox and benznidazole, which have been in use since the late 1960s and are compromised by adverse side reactions and low efficacy in chronic disease [3], [4]. A need for drugs with more consistent efficacy and less toxicity is manifest.

With an essential requirement for ergosterol [5] and an inability to survive solely on cholesterol salvaged from the host, *T. cruzi* is vulnerable to inhibitors of the sterol biosynthesis enzyme 14α-demethylase (CYP51) [6], [7]. Disruption of CYP51 results in alteration in the ultrastructure of several organelles, decline of endogenous sterols in the parasites, and an accumulation of various 14α-methyl sterols with cytostatic and cytotoxic consequences [8]. The broad spectrum antifungal drug posaconazole (Noxafil; Schering-Plough) [9], which targets CYP51, is poised for clinical trials against *T. cruzi* [6], [10], [11]. Posaconazole is capable of inducing parasitological cure in a murine model of both acute and chronic Chagas Disease, curing between 50–100% of animals in the acute phase of infection, and 50–60% of animals chronically infected [7], [11]. However, the high manufacturing cost of posaconazole and the requirement for administration via oral suspension simultaneously with a fatty meal or nutritional supplement to enhance absorption may limit its use in treating chronic *T. cruzi* infections [12]. The search for CYP51-specific compounds that are easier to synthesize and better absorbed upon oral administration continues [13]–[17].

To rationalize protein-ligand interactions for new inhibitors in *T. cruzi*, homology modeling based on the x-ray structure of CYP51 from *Mycobacterium tuberculosis* (CYP51_Mt_) [18]–[20] has been used [14], [15], [17]. But CYP51_Mt_ has only 27% sequence identity to the *T. cruzi* enzyme and is unusually exposed to the bulk solvent at the substrate binding site. This structural peculiarity largely excludes the functionally important BC-loop from protein-inhibitor interactions and thus limits the utility of CYP51_Mt_ as a model for a Chagas Disease target. The CYP51 BC-loop residue 105 (numbering according to *T. cruzi* and *T. brucei* CYP51) is indispensable in the discrimination of the species-specific sterol substrates in *T. cruzi* and *T. brucei* [19], [21]. Also, a critical mutation hot spot [22], the well conserved BC-loop residue Y116 was reported to be involved in fungal drug resistance, inhibitor binding, and the catalytic function of CYP51 in *Candida albicans* (Y132, according to *C. albicans* numbering) [22]–[27], *Histoplasma capsulatum* (Y136, according to *H. capsulatum* numbering) [28], and in the causative agents of zygomycosis in humans, *Rhizopus oryzae* and *Absidia corymbifera* [29]. It may therefore play a similar role in *T. cruzi*.

Here we report the crystal structures for the CYP51 target in *T. cruzi* (CYP51_Tc_) (resolutions 2.35 Å and 2.27 Å) and that of the closely related CYP51 ortholog from *Trypanosoma brucei* (CYP51_Tb_) (resolutions 2.7 Å and 2.6 Å), each bound to an anti-fungal triazole drug, either fluconazole or posaconazole. *T. brucei* is a protozoan parasite closely related to *T. cruzi* [30] and the agent of another lethal tropical disease, African Sleeping Sickness. In contrast to *T. cruzi* and *Leishmania spp.*, it is not clear if the sterol biosynthesis pathway can be targeted in *T. brucei*. Each parasite has a different life-cycle and different sterol requirements. Although the insect (procyclic) form of *T. brucei* can undertake *de novo* sterol biosynthesis, the latter is apparently suppressed in the bloodstream form in the mammalian host, which is supported by receptor-mediated endocytosis of host low-density lipoproteins that carry phospholipids and cholesterol esters [31]. Nevertheless, CYP51_Tc_ and CYP51_Tb_ do share 83% sequence identity, a fact which has been crucial for successfully determining their crystal structures and makes it possible to extrapolate structural features learned from one enzyme toward the other. Furthermore, the *Leishmania* CYP51 are 72–78% identical to that of *T. cruzi and T. brucei*, so they too can now be modeled to facilitate drug discovery and development.

## Materials and Methods

### Design of expression vectors

By trial-and-error we empirically identified the protein N-terminal modification that eventually led to CYP51 crystals of sufficient quality to determine the x-ray structure. To improve our chances for success, we did the work in parallel on CYP51 proteins from *Trypanosoma cruzi* and *Trypanosoma brucei*. Five different expression vectors were designed in this work for each CYP51 ortholog to eliminate a stretch of hydrophobic residues which presumably mediate association of the proteins with the endoplasmic reticulum (ER). In their place we introduced hydrophilic or charged sequences at the N-terminus (Table 1). His_6_-tag (CYP51_Tc_) or His_8_-tag (CYP51_Tb_) was introduced at the C-terminus to facilitate purification. Coding sequences were sub-cloned between the NdeI and HindIII restriction cloning sites of the pCWori vector [32] and in this form used to transform *Escherichia coli* strain HMS174(DE3). The original coding sequence for CYP51_Tb_ contained an internal NdeI site at 345 bp which was silenced by QuickChange site-directed mutagenesis (Stratagene) using forward GGGGTT**GCC**TATGCTGCC and reverse CCCCAA**CGG**ATACGACGG PCR primers. DNA amplification reaction: 5 min at 94°C, annealing for 1 min at 50–60°C, extension for 1.5 min at 72°C, for 30 cycles, followed by extension for 10 min at 72°C. The highest expression levels were achieved and the best crystals were obtained from the expression constructs modified by replacing the first 21 residues upstream of K22 with the fragment MAKKKKK. Subsequently, based on the analysis of the packing interactions in the crystal, three consecutive glutamate residues, E249-E251, were replaced in CYP51_Tb_ with alanine by site-directed mutagenesis (Stratagene) using forward GCGCG**GCTGCTGCT**GTCAACAAGGACAGC and reverse GCGCG**AGCAGCAGC**CTTTCGAGCAATGAT PCR primers. DNA amplification reaction: 5 min at 94°C, annealing for 1 min at 45–65°C, extension for 1.5 min at 72°C, for 35 cycles, followed by extension for 10 min at 72°C. This CYP51_Tb_ variant was used to generate the CYP51_Tb_-posaconazole crystals. The identity of all resulting vectors was confirmed by DNA sequencing.

**Table 1:** Design and analysis of the expression vectors.

Protein	Truncation1<\emph> 10 20 30 40	Yield	Crystals
CYP51_Tc_WT	MFIEAIVLGLTALILYSVYSVKSFNTTRPTDPPVYPVTVP	N/A	No
CYP51_Tc_#1	MAKKTSSKGKL PPVYPVTVP	4 mg/l	Yes
CYP51_Tc_#2	MA PPVYPVTVP	N/A	No
CYP51_Tc_#3	MA KSFNTTRPTDPPVYPVTVP	N/A	No
**CYP51_Tc_#4**	**MAKKKKK KSFNTTRPTDPPVYPVTVP**	**75 mg/l**	**Yes**
CYP51_Tc_#5	MAKKKKK PPVYPVTVP	5 mg/l	No
CYP51_Tb_WT	MLLEVAIFLLTALALYSFYFVKSFNVTRPTDPPVYPVTVP	N/A	No
CYP51_Tb_#1	MAKKTSSKGKL PPVYPVTVP	1 mg/l	No
**CYP51_Tb_#2**	****MAKKKKK KSFNTTRPTDPPVYPVTVP****	**3 mg/l**	**Yes**
CYP51_Tb_#3	MAKKTSSDEVDEVDEV DPPVYPVTVP	N/A	No
CYP51_Tb_#4	MADEVDEVDEV DPPVYPVTVP	N/A	No
CYP51_Tb_#5	MA PPVYPVTVP	N/A	No

Highlighted in bold are the constructs which led to the corresponding x-ray structures.

### Protein expression and purification

#### CYP51_Tc_

One liter of Terrific Broth medium supplemented with 1 mM thiamine, 100 µg/ml ampicillin and trace elements was inoculated with 15 ml of the night culture. Growth continued at 37°C and 240 rpm agitation until OD_590_ reached 1.0. CYP51_Tc_ expression was induced by the addition of isopropyl-β-D-thiogalactopyranoside (IPTG, final concentration 0.25 mM) and δ-aminolevulinic acid, a precursor of heme biosynthesis (final concentration 1 mM). Following induction, temperature was decreased to 28°C and agitation to 180 rpm. After 40 hours the cells were harvested and lysed by sonication. Insoluble material was removed from crude extract by centrifugation (45 min at 35,000 rpm). The supernatant was subjected to a series of chromatographic steps, including nickel-nitrilotriacetic acid (Ni-NTA) agarose (QIAGEN), followed by Q-Sepharose (Amersham Biosciences) in flow-through regime, then by S-Sepharose (Amersham Biosciences). From the S-Sepharose, protein was eluted in a 0.2 to 1.0 M NaCl gradient and observed by means of 12% SDS-PAGE to be virtually homogeneous. Fractions containing P450 were combined, concentrated using a Centriprep concentrating device (Millipore), and stored at −80°C. Twenty mM Tris-HCl, pH 8.0, 10% glycerol, 0.5 mM EDTA, and 1 mM DTT were maintained throughout all chromatographic steps.

#### CYP51_Tb_

In contrast to CYP51_Tc_, CYP51_Tb_ yield was significantly improved via co-expression of the *E. coli* chaperones GroES and GroEL ecoded by the pGro7 plasmid (Takara) co-transformed into the HMS174(DE3) strain. Double transformants were selected on both ampicillin and chloramphenicol containing agar plates. One liter of Terrific Broth medium supplemented with 1 mM thiamine, 100 µg/ml ampicillin, 40 µg/ml chloramphenicol and trace elements was inoculated with 10 ml of the night culture. Growth continued at 37°C and 240 rpm agitation until OD_590_ reached 0.2. Expression of chaperones from the pGro7 vector was induced with 0.2% arabinose. Growth continued at 26°C and 180 rpm until OD_590_ reached 0.8. Then CYP51_Tb_ expression was induced by the addition of isopropyl-β-D-thiogalactopyranoside (IPTG), final concentration 0.4 mM; δ-aminolevulinic acid, final concentration 1 mM, was also added at this time. Following induction, temperature was decreased to 21°C. After 44 hours the cells were harvested and lysed by sonication. Purification was conducted as described for CYP51_Tc_.

Both proteins were characterized by UV-vis spectroscopy. For crystallization, protein concentration was determined at 450 nm from the difference spectra between the CO-bound ferrous and water-bound ferric forms, with an extinction coefficient of 91,000 M^−1^cm^−1^ [33].

### Crystallization and data collection

Screening of crystallization conditions was routinely performed following purification of protein variants using commercial screening kits available in high throughput screening format (Hampton Research), a nanoliter drop-setting Mosquito robot (TTP Labtech) operating with 96-well plates, and a hanging drop crystallization protocol. Optimization of crystallization conditions, if required, was carried out manually in 24-well plates at 23°C. Proteins were from 1.0–1.8 mM frozen stocks in 20 mM Tris-HCl, pH 7.2 (CYP51_Tb_) or pH 8.0 (CYP51_Tc_), 10% glycerol, 0.5 mM EDTA, and 1 mM DDT. The CYP51_Tb_ triple mutant E249A/E250A/E251A was used to obtain CYP51_Tb_-posaconazole crystals. Prior to crystallization proteins were diluted to 0.1–0.2 mM by mixing with 50 mM potassium phosphate at appropriate pH, supplemented with 0.5 mM (CYP51_Tb_) or 0.1 mM (CYP51_Tc_) fluconazole. Dilution in the absence of fluconazole or phosphate caused fast precipitation of protein samples. Posaconazole was prepared as 10 mM stock solution in DMSO and has been used at final concentration of 0.2 mM. Protein-posaconazole mix was incubated at 4°C for one hour prior to crystallization. Crystals of CYP51_Tb_–fluconazole complex grew from 15% ethylene glycol and 0–3% acetonitrile. Crystals of CYP51_Tb_–posaconazole complex grew from 6% PEG 4000, 2% tacsimate, pH 8.0, and 2% DMSO. Crystals of CYP51_Tc_–fluconazole grew either from 40% polypropylene glycol 400 and 0.1 M Tris-HCl, pH 6.0 (PDB ID 2WUZ), or from 25% PEG 4000 and 0.1 M Bis-Tris, pH 5.5 (PDB ID 2WX2), the latter being harvested directly from the Mosquito 200-nl drop. Prior to data collection, the crystals were cryo-protected by plunging them into a drop of reservoir solution supplemented with 20–24% ethylene glycol or 20% glycerol, and flash-frozen in liquid nitrogen.

All native and two-wavelength anomalous dispersion x-ray diffraction data were collected at 100–110 K at beamline 8.3.1, Advanced Light Source, Lawrence Berkeley National Laboratory, USA. Anomalous diffraction data were collected from one CYP51_Tb_ crystal at two wavelengths, one corresponding to the median between the Fe peak and the inflection point and the other at 375 eV higher (Table 2). Data indexing, integration, scaling, phasing, and density modification were conducted using the ELVES automated software suite [34] (Tables 2 and 3).

**Table 2:** Fe anomalous dispersion data collection and phasing statistics.

Protein	CYP51_Tb_	
Ligand	Fluconazole	
**Data collection**		
Space group	P3_1_21	
Cell dimensions		
* a*, *b*, *c* (Å)	106.4, 106.4, 99.8	
α,β, γ (°)	90, 90, 120	
Molecules in AU	1	
	*Peak/Inflection*	*Remote*
Wavelength	1.7393	1.6531
Resolution (Å)	3.2	3.2
*R*_sym_ or *R*_merge_ (%)	6.5 (–)^1^	6.2 (–)
*I*/σ*I*	21.1 (2.3)	22.4 (2.9)
Completeness (%)	100 (93.4)	100 (99.1)
Redundancy	18.8 (9.5)	20.2 (13.0)
**Phasing**		
Resolution range	3.2–67.7	
No. of used sites	1	
Phasing power	0.38	0.36
Figure of merit	0.26	
After density modification	0.85	

1 Values in parentheses are for highest-resolution shell; R_sym_ is meaningless when the individual spot I/σI value is below 1.

**Table 3:** Data collection and refinement statistics.

	Native			
Protein	CYP51_Tc_	CYP51_Tc_	CYP51_Tb_	CYP51_Tb_
Ligand	Fluconazole	Fluconazole	Fluconazole	Posaconazole
PDB ID	2WX2	2WUZ	2WV2	2X2N
**Data collection**				
Space group	P2_1_	P2_1_	P3_1_21	C2
Cell dimensions				
* a*, *b*, *c* (Å)	70.0, 101.5, 74.7	74.9, 92.6, 78.3	106.2, 106.2, 99.7	199.9, 114.5, 138.1
α,β,γ (°)	90, 111.63, 90	90, 102.1, 90	90, 90, 120	90, 131.8, 90
Molecules in AU	2	2	1	4
Wavelength	1.1159	1.1159	1.1159	1.1159
Resolution (Å)	2.27	2.35	2.7	2.6
*R*_sym_ or *R*_merge_ (%)	9.0 (42.0)^1^	11.0 (50.0)	10.7 (–)^2^	8.5 (76.9)
*I*/σ*I*	8.5 (2.4)	7.4 (1.5)	17.0 (2.8)	9.2 (1.7)
Completeness (%)	100.0 (55.7)	93.6 (70.8)	100.0 (100.0)	99.8 (100.0)
Redundancy	3.6 (2.4)	3.4 (2.4)	44.1 (44.5)	3.9 (3.9)
**Phasing**				
Resolution range				
No. of used sites				
Phasing power				
Figure of merit				
After density modification				
**Refinement**				
No. reflections	38067	38792	17043	71168
*R*_work_/*R*_free_ (%)	19.3/27.3	21.7/27.5	21.0/27.4	19.1/26.4
No. atoms				
Protein	6954	6994	3433	14048
Heme	86	86	43	172
Ligand	44	44	22	204
Water	429	179	15	253
Mean B value	23.7	44.6	45.8	53.9
*B*-factors				
Protein	23.8	44.9	37.2	54.2
Heme	15.5	41.6	65.0	47.7
Ligand	32.1	47.0	93.1	50.7
Water	25.3	43.5	36.6	46.4
R.m.s deviations				
Bond lengths (Å)	0.016	0.019	0.018	0.016
Bond angles (°)	1.7	1.9	1.8	1.7

1 Values in parentheses are for highest-resolution shell.

2 R_sym_ is meaningless when the individual spot I/σI value is below 1.

### Structure determination and refinement

CYP51_Tb_–fluconazole data processed in P3_1_21 with R_merge_ of 6.5% allowed for location of a single Fe atom. Initial phases with an overall figure of merit of 0.26 were improved by solvent flattening (mean figure of merit 0.85 after solvent flattening) to provide an interpretable electron density map (Table 2). Automated model building using BUCCANEER [35] placed the polyalanine backbone for 84% of the residues in the asymmetric unit. The remaining residues were built manually with COOT [36], alternated with TLS and positional refinement using REFMAC [37], [38]. The structure was refined to 3.2 Å with the R and R_free_ values of 32.0% and 38.0%, respectively. Although showing up largely as a polypeptide backbone at low resolution, this *T. brucei* structure served as a search model for molecular replacement in determining the x-ray structure for CYP51_Tc_ using 2.35 Å native data processed as P2_1_ with R_merge_ of 11%. Two CYP51_Tc_ molecules were placed in an asymmetric unit. Manual model building with COOT [36] alternated with TLS and positional refinement using REFMAC [37], [38] resulted in the final CYP51_Tc_ structure with the R and R_free_ =  values of 21.7% and 27.5% and the Ramachandran statistics of 93.8% residues in preferred regions, 5.2% in allowed regions, and 1% (9 residues) outliers, as calculated by COOT. NCS restrains were applied at all stages of the refinement. The refined CYP51_Tc_ structure was used as a starting model against both the 2.27 Å native data for CYP51_Tc_ and 2.7 Å native data for CYP51_Tb_, which allowed the majority of the CYP51_Tb_ side chains to be built in. At that time, refinement of CYP51_Tb_ converged with R and R_free_ of 21.0% and 27.4%, respectively. Ramachandran statistics indicate 91.2% residues in preferred regions, 5.9% in allowed regions, and 2.9% (13 residues) outliers. Refinement of 2.27 Å CYP51_Tc_ data converged with R and R_free_ of 19.3% and 27.3%, respectively, and the Ramachandran statistics of 95.6% residues in preferred regions, 3.5% in allowed regions, and 0.9% (9 residues) outliers.

Analysis of the crystallographic symmetry packing interactions in the CYP51_Tb_-fluconazole complex revealed contacts between the triplets of glutamate residues D249-D251 situated in the GH-loop. To reduce electrostatic repulsion, all three residues were replaced with alanine. Although this modification did not improve resolution of the CYP51_Tb_-fluconazole crystals, the triple mutant was more amenable to co-crystallization with posaconazole. The CYP51_Tb_ coordinates refined to 2.7 Å served as a search model to determine CYP51_Tb_-posaconazole structure using 2.6 Å native data processed as C2 with R_merge_ of 8.5%. Four protein molecules were placed in an asymmetric unit. Refinement converged with R and R_free_ of 19.1% and 26.4%, respectively and the Ramachandran statistics of 95.3% residues in preferred regions, 4.0% in allowed regions, and 0.7% (13 residues) outliers. In all structures, side chains not visible in the density were modeled as alanine (Table 3).

### Molecular docking

**Figure 1:**
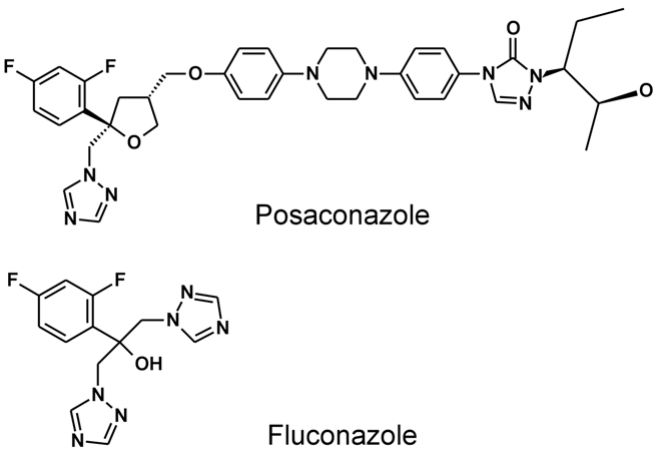
Chemical structures of posaconazole and fluconazole.

Binding of posaconazole to CYP51_Tc_ was predicted by molecular docking using the 2WUZ structure. Docking was carried out using GLIDE (version 5.0) [39]. The docking protocol was validated by re-docking of fluconazole, which reproduced the binding mode observed in the crystal structure. The protein was initially prepared by the Protein Preparation Wizard module using default options. Hydrogen atoms were added to the complex structure, followed by a restrained minimization using the OPLS2005 force field. The Receptor Grid Generation module was then employed to prepare a rigid receptor grid centered at M360, which contains the entire binding tunnel of the energy minimized complex, for subsequent docking. The three-dimensional structure of posaconazole was generated by the Ligprep module with the OPLS2005 force field. Computational docking was performed using GLIDE in standard precision (SP) mode, and binding affinities were estimated as GLIDE score. The characteristic coordination between the heme group and the ligand was modeled by applying a constraint at the Fe^3+^ ion of the heme group that imposed interaction with one of the nitrogen atoms from the ligand's triazolyl ring. Since posaconazole (molecular weight = 700.8 g/mol) is significantly larger and longer than fluconazole (molecular weight = 306.3 g/mol) (Fig. 1), the van der Waals radii of the ligand were softened by a scaling factor of 0.6 in the initial docking calculation, which predicted two binding poses with similar Glide scores of −9.23 and −9.60. The binding model was further refined by relaxing the binding tunnel in the presence of posaconazole. Side chains of residues within 4 Å from the docked posaconazole were optimized by performing side-chain refinement with Prime (version 2.0) [40]. The resulting complex was used to re-dock posaconazole with van der Waals radii scaled by the default value of 0.8. Consistent with the initial calculations, the second-round docking also predicted the same binding orientations with favorable and similar GLIDE scores (pose 1 = −11.07; pose 2 = −10.70).

### Accession codes

Protein Data Bank: coordinates and structure factors have been deposited with accession codes 2WUZ, 2WX2, 2WV2 and 2X2N.

## Results and Discussion

### Protein design and determination of the x-ray structures

**Figure 2:**
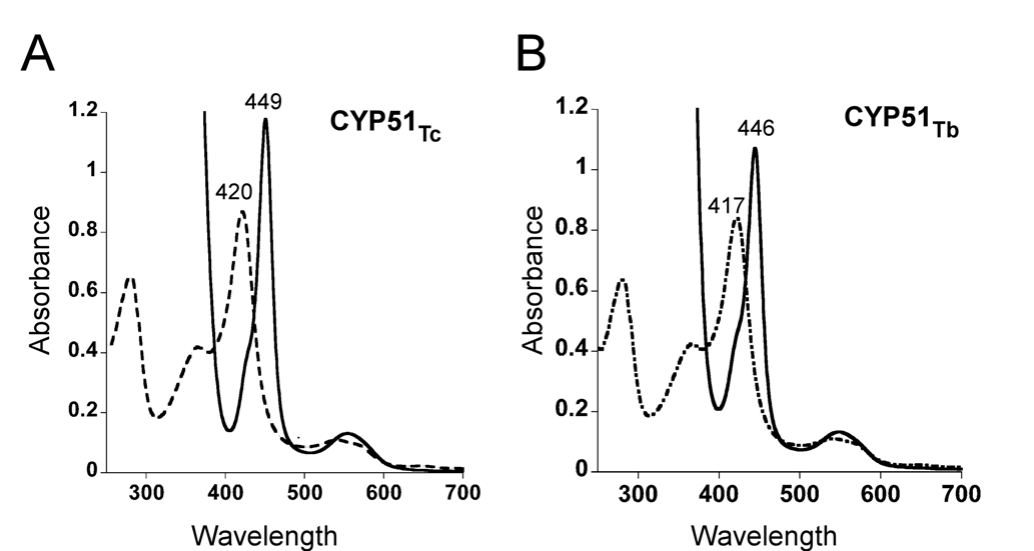
Spectral characterization of CYP51_Tc_ and CYP51_Tb_. Soret and visible regions of the CYP51_Tc_ (**A**) and CYP51_Tb_ (**B**) spectra are shown. The ferric protein (dashed trace) was reduced with sodium dithionite to a ferrous form (solid trace) in the presence of CO. The spectra were recorded at room temperature in a 1 ml quartz cuvette containing 1 µM CYP51 in 10 mM Tris-HCl, pH 7.5, and 10% glycerol using a Cary UV-visible scanning spectrophotometer (Varian). CYP51_Tc_ has a Soret maximum at 420 nm which upon reduction with sodium dithionite and CO binding shifts to 449 nm (**A**). CYP51_Tb_ has a Soret maximum at 417 nm which upon reduction and CO binding shifts to 446 nm (**B**).

By trial-and-error, the highest expression levels and best crystals for both CYP51_Tc_ and CYP51_Tb_ were obtained from the expression constructs modified by replacing the first 21 residues upstream of K22 with the highly positively charged fragment MAKKKKK (Table 1). The triple E249A/E250A/E251A CYP51_Tb_ mutant was based upon this N-terminally modified construct. The UV-vis spectra of purified proteins revealed features characteristic for homogeneous and normally folded P450 (Fig. 2). We first determined the crystal structure for CYP51_Tb_ using anomalous dispersion of the heme iron. Although largely a backbone trace at 3.2 Å resolution, this structure served as a search model for molecular replacement in determining the CYP51_Tc_–fluconazole structure at 2.35 Å, which was used as a search model against the 2.27 Å CYP51_Tc_–fluconazole data to reveal an alternative conformation of fluconazole bound in the active site. This same CYP51_Tc_ structure was used as a model against the 2.7 Å CYP51_Tb_–fluconazole data. Refined to 2.7 Å CYP51_Tb_ coordinates subsequently served as a search model for determining the 2.6 Å CYP51_Tb_–posaconazole structure.

### Overall crystal structures of CYP51

**Figure 3:**
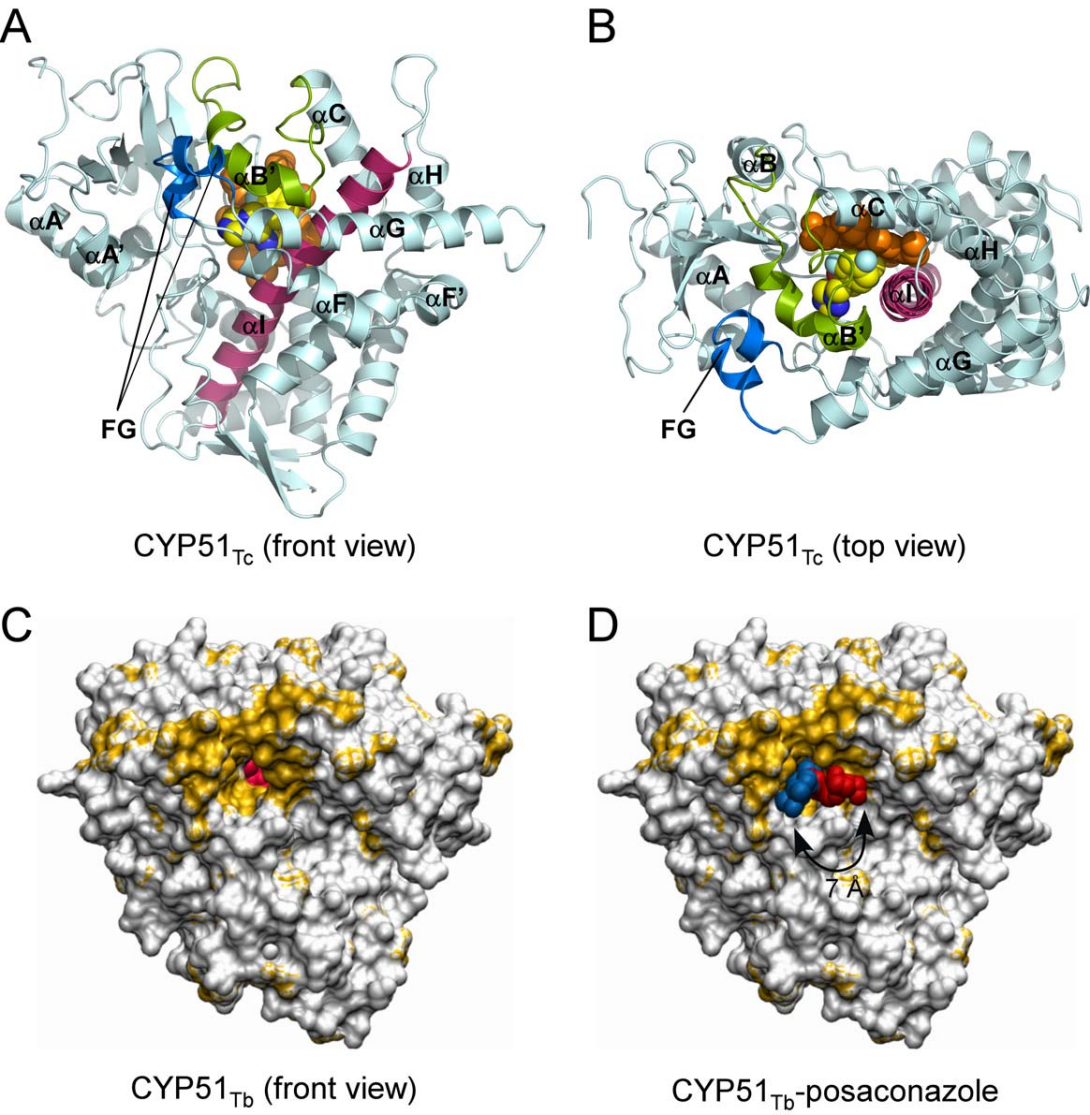
Overall structures of CYP51. **A, B.** Fluconazole-bound CYP51_Tc_ with selected α-helices labeled (PDB ID Code 2WUZ). **A.** Distal protein surface with respect to heme. **B.** Image is rotated ∼90° toward viewer. Protein backbone is depicted by cyan ribbon with the I-helix highlighted in magenta, BC-region in green, FG-region in blue. Heme (orange) and fluconazole are depicted by spheres. Fluconazole color scheme: carbon yellow, oxygen red, nitrogen blue, fluorine cyan. Images were prepared using PYMOL [58] unless indicated otherwise. **C, D.** CYP51_Tb_ in surface representation with the hydrophobic residues colored in golden yellow. In **C**, posaconazole is omitted for clarity, heme prosthetic group (pink) shows through the hydrophobic tunnel entrance. In **D**, two overlaid posaconazole conformers are shown protruding out of the tunnel opening. Bent conformer (chain B) is in red, extended conformer (chain D) is in blue. Images were generated using VMD program [59].

**Figure 4:**
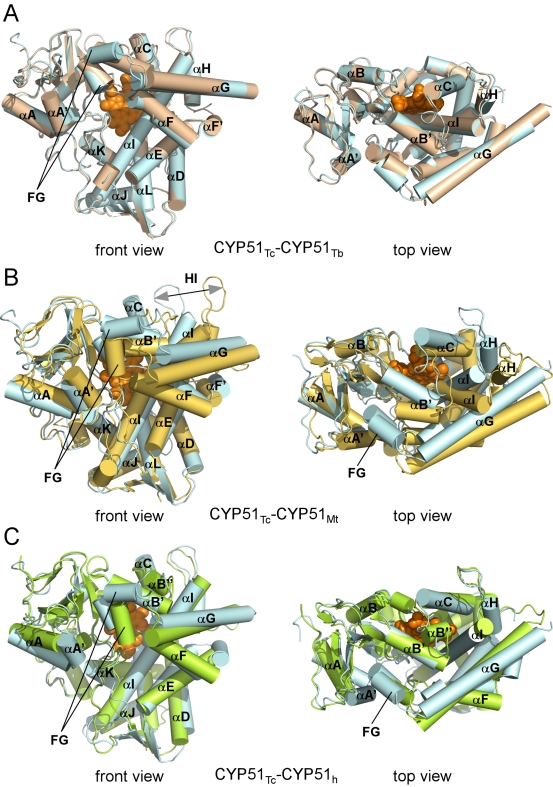
Comparison between CYP51 from different phyla. **A.** CYP51_Tc_ (cyan in **A**, **B** and **C**, PDB ID Code 2WUZ) and CYP51_Tb_ (wheat, PDB ID Code 2WV2) superimposed with r.m.s.d. of 0.89 Å. Helices are represented by labeled cylinders. Fluconazole is omitted for clarity. **B.** CYP51_Tc_ and CYP51_Mt_ (golden yellow, PDB ID Code 2VKU) superimposed with r.m.s.d of 1.83 Å. **C.** CYP51_Tc_ and CYP51_h_ (lemon green, PDB ID Code: 3I3K) superimposed with r.m.s.d of 1.45 Å. In each panel, distal surface is shown on the right. Image on the left is rotated ∼90° toward viewer.

CYP51_Tc_ and CYP51_Tb_ have a common P450 protein fold characterized by the sets of the α-helices and β-sheets highlighted in Fig. 3A, B. The *T. cruzi* and *T. brucei* structures superimpose with r.m.s.d. of 0.89 Å for Cα atoms, with the most pronounced differences in the region encompassing the F and G helices and the loop between them (Fig. 4A). By contrast, *Trypanosoma* CYP51 enzymes do not superimpose nearly as well with bacterial CYP51_Mt_ (r.m.s.d. of 1.83 Å) (Fig. 4B), being more similar to their human counterpart (CYP51_h_), based on both backbone similarity (r.m.s.d. of 1.45 Å) and solvent exposure at the active site (Fig. 4C). All three eukaryotic enzymes lack the extreme bending of the I-helix that is associated with CYP51_Mt_, resulting in their active sites being more isolated from the bulk solvent. The structured BC-region in *Trypanosoma* CYP51 includes the B'-helix encompassed by the short η-helices blocking access to the active site from the bulk solvent. Seven residues from the BC-region, V102, Y103, I105, M106, F110, A115 and Y116, are part of the active site in CYP51_Tc_ and CYP51_Tb_, which is consistent with our previous observation that a series of CYP51 inhibitors reported elsewhere [16] have higher binding affinities toward *Trypanosoma* CYP51 compared to CYP51_Mt_, where these residues do not participate in the active site due to the “open” conformation of the loop. The two CYP51_Tc_–fluconazole structures reported here superimpose with r.m.s.d of 0.68 Å, revealing some conformational differences in the F-helix and the BC-loop, which may account for the distinct fluconazole binding modes and result in re-packing of protein molecules in the crystal lattice (Table 3).

In the CYP51_Tb_-posaconazole structure, four protein molecules in the asymmetric unit superimpose with the r.m.s.d. within of 0.5 Å, revealing virtually no conformational variations. However, posaconazole samples two distinct conformations due to the long tail swinging ∼7–8 Å in the hydrophobic mouth of the substrate binding tunnel (Fig. 3B). The entrance to the tunnel is marked by a patch of the hydrophobic residues (colored yellow in Fig. 3B), which apparently guide access of the sterol substrates to the active site.

### Fluconazole binding in the active site

**Figure 5:**
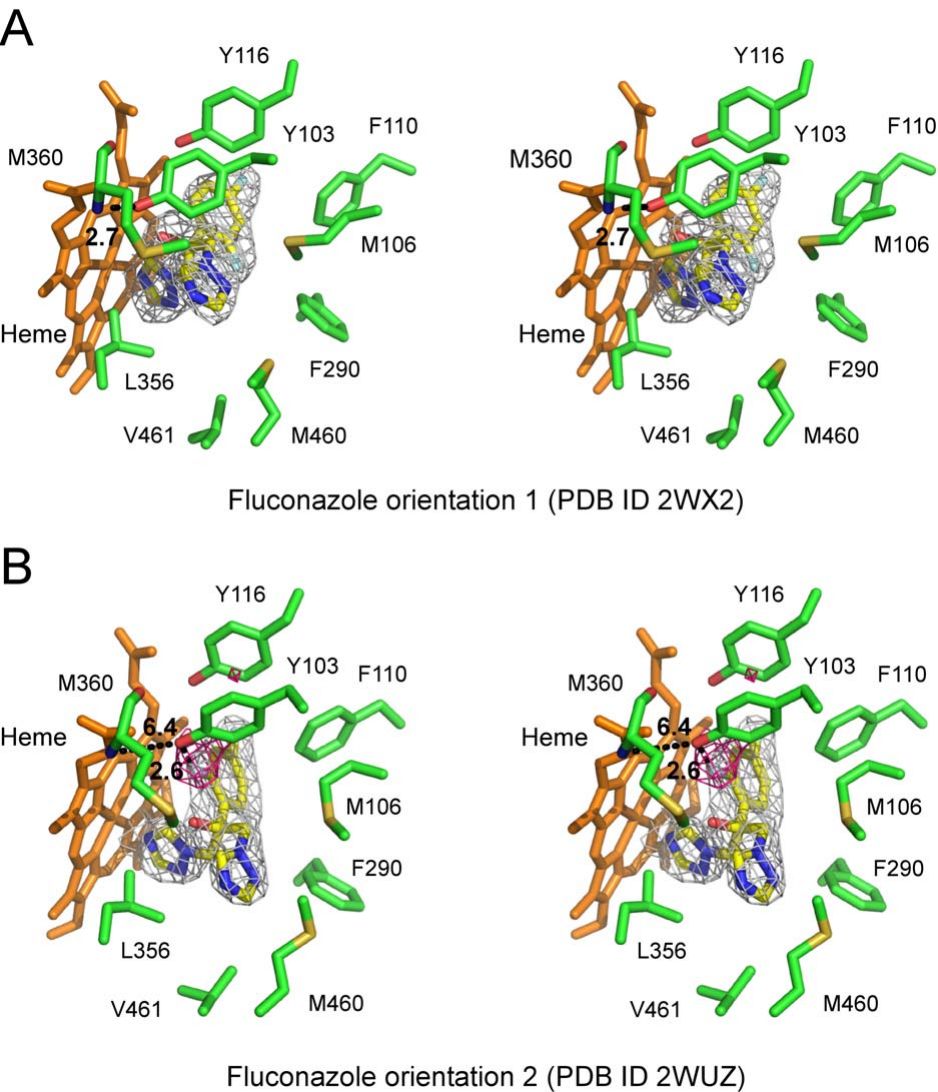
Fluconazole binding in CYP51_Tc_. **A, B.** Stereoscopic view of CYP51_Tc_ with fluconazole bound in active site. Side chains of the residues within 4 Å of fluconazole are in green. For clarity, A287, A291 and T295 are omitted. Main chain atoms are shown for M360. Fluconazole color scheme as in **Fig. 3**. Fragments of 2F_o_-F_c_ electron density map calculated with the fluconazole coordinates omitted from the input file are shown as grey wire mesh. Chain A has been used in both structures to generate the images. **A.** Fluconazole orientation 1 in 2WX2 structure; OH-group of Y103 H-bonds to the amide nitrogen of M360. **B.** Fluconazole orientation 2 in the 2WUZ structure; peak in the positive F_o_-F_c_ map (pink mesh) calculated with 2,4-difluorophenyl ring in flipped orientation superimposes with the 2-fluorine H-bonding to Y103.

**Figure 6:**
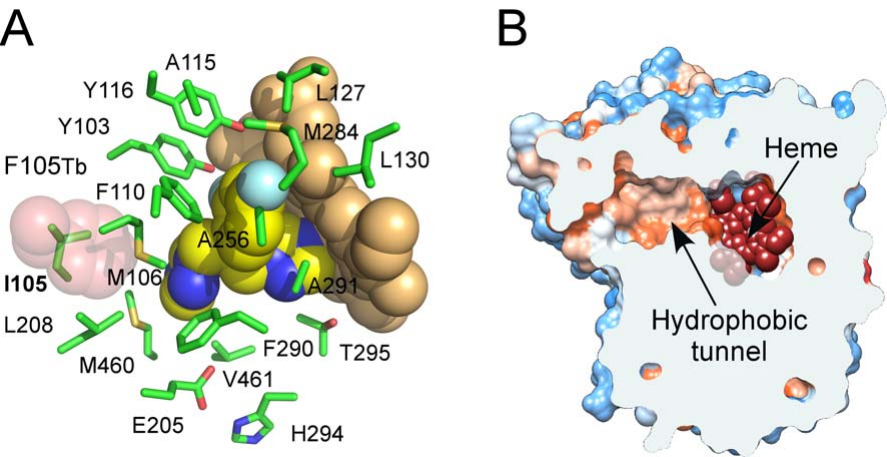
Hydrophobic tunnel. **A.** Fluconazole in orientation 2 bound to CYP51_Tc_. Fluorinated edge of the 2,4-difluorophenyl ring faces heme macrocycle. Fluconazole and heme are shown as van der Waals spheres; residues within 7 Å of fluconazole as sticks. Color scheme for heme and fluconazole as in **Fig. 3**. F105 superimposed from CYP51_Tb_ is shown as semitransparent pink spheres. **B.** Front view of CYP51_Tc_ clipped by plane (cyan) through substrate binding tunnel. Hydrophobic areas are orange, hydrophilic areas blue. Heme at end of tunnel: with van der Waals spheres in red. Fluconazole is removed for clarity. Image was prepared using CHIMERA [60].

**Figure 7:**
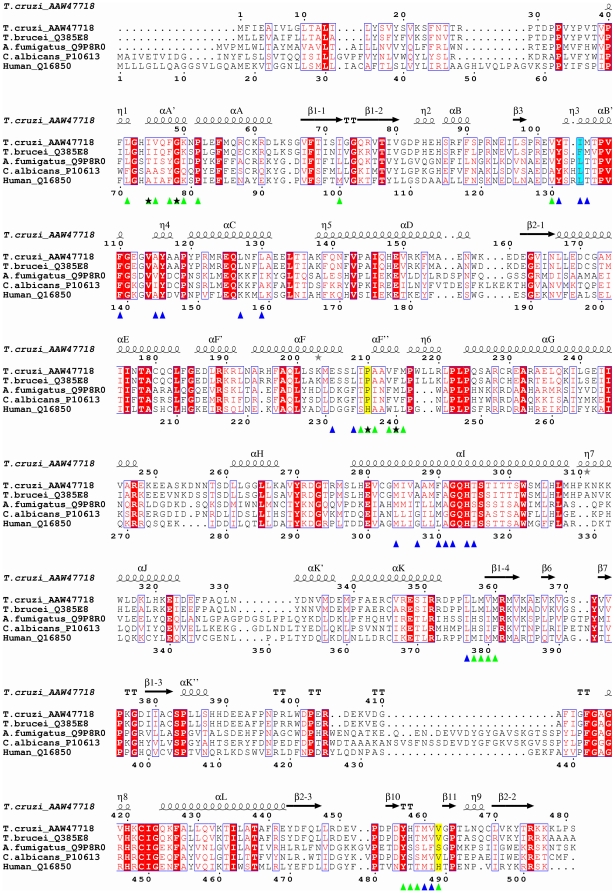
Sequence alignments between host and pathogen CYP51. Sequence alignments between CYP51 from *Trypanosoma cruzi*, *Trypanosoma brucei, Aspergillus fumigatus*, *Candida albicans* and human. Accession numbers of the proteins in the Swiss-Prot/TrEMBL (http://us.expasy.org/sprot) and NCBI (http://www.ncbi.nlm.nih.gov/) databases are given next to the name of the protein. Alignments were performed using CLUSTALW program online [61]. The figure was generated using ESPript [62]. The secondary structure annotation and residue numbering at the top correspond to CYP51_Tc_, residue numbering at the bottom corresponds to human CYP51. The α-helices are labeled with capital letters according to generally accepted P450 nomenclature. The β-strands of large β-sheets are labeled with dashed numbers. Sequential numbers are used to label short two-residue β-strands. Residues within 7 Å of fluconazole are labeled with blue triangles. Additional residues constituting the hydrophobic tunnel are labeled with green triangles. Human H236 and H489 and the corresponding residues in the pathogenic species are highlighted in yellow. Residues corresponding to CYP51_Tc_ I105 are highlighted in cyan. Mutation hot spots at the tunnel opening are marked with black stars. Gray stars highlight residues in alternate conformations.

As expected, fluconazole is bound in the active site by coordination to the heme iron via the aromatic nitrogen atom of a triazole ring and by multiple van der Waals and aromatic stacking interactions (Fig. 5). All residues within 7 Å of fluconazole (Fig. 6A) are labeled with blue triangles in Fig. 7. The 2,4-difluorophenyl moiety is enclosed in the pocket formed by the heme macrocycle, the aromatic side chains of Y103, F110, Y116 (BC-loop) and F290 (I-helix), and aliphatic side chains M106, A287 and A291. Although fluconazole occupies the same pocket in both CYP51_Tc_ structures, it adopts two conformations that differ by the 180° flipping of the 2,4-difluorophenyl moiety. Orientation 1 is observed both in the 2.27 Å CYP51_Tc_–fluconazole structure reported in this work (PDB ID 2WX2) (Fig. 5A) and in the CYP51_Mt_-fluconazole complex reported elsewhere (PDB ID 1EA1) [18]. The same conformation is adopted by the 2,4-difluorophenyl ring of posaconazole in the CYP51_Tb_-posaconazole complex in all four molecules in the asymmetric unit. In orientation 1, Y103 makes a 2.7 Å H-bonding contact to the main chain amide group of M360. A 180° flipped orientation of the ring, orientation 2, is observed in the 2.35 Å CYP51_Tc_–fluconazole structure (PDB ID 2WUZ) (Fig. 5B). As evidenced by the residual F_o_-F_c_ electron density map calculated for the orientation 1 (pink mesh in Fig. 5B), the 2-fluoro substituent of the fluconazole difluorophenyl ring in 2WUZ must point toward the heme macrocycle. A 2.6 Å H-bonding contact between the 2-fluoro substituent and the hydroxyl group of Y103 may help to stabilize orientation 2, which appears to be less sterically favorable than orientation 1.

Perhaps both ring conformations co-exist in the CYP51_Tc_-fluconazole complex, possibly correlated with the conformation of the BC-loop which affects H-bonding pattern of Y103. In orientation 1, the H-bond between the 2-fluoro substituent and Y103 is broken due to the 3.5 Å reorientation of Y103 toward M360 resulting in the 2.7 Å H-bond to its amide nitrogen and in the flipping of the 2,4-difluorophenyl ring into a sterically more favorable orientation with the fluorinated edge facing away from the heme macrocycle (Fig. 5B). Crystallization conditions may have served to shift the equilibrium by stabilizing one of these states. The entire fluconazole molecule is shifted about 1.5 Å between the two CYP51_Tc_ structures, which may be related to the low efficacy of this drug against *T. cruzi*. Given that the CYP51_Tb_ active site is virtually identical to that of *T. cruzi*, the same equilibrium would be expected to occur in *T. brucei*. However, we could not observe this phenomenon as CYP51_Tb_-fluconazole complex has been co-crystallized under a single set of conditions with one molecule in the asymmetric unit.

### Hydrophobic tunnel

**Figure 8:**
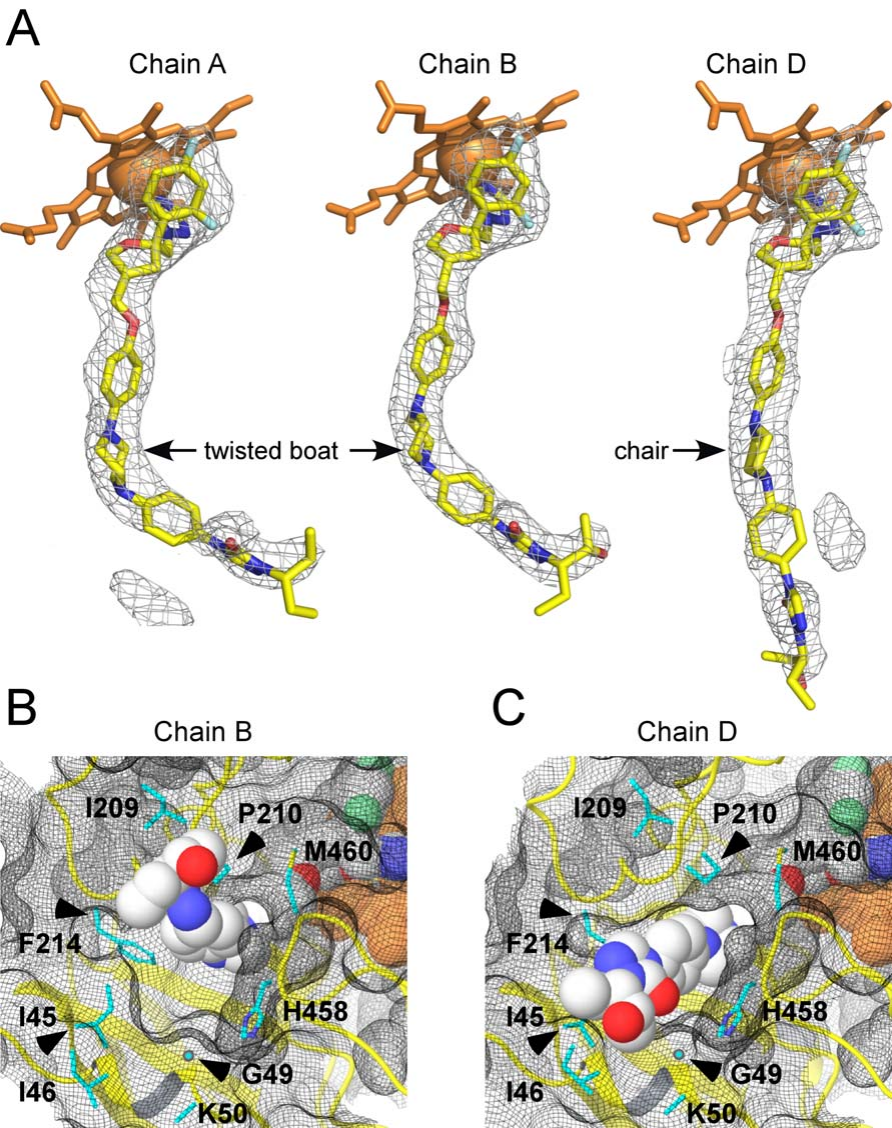
Posaconazole conformation. **A.** Fragments of the F_o_-F_c_ electron density map (grey mesh) in chains A, B and D calculated with the posaconazole coordinates omitted from the input file. **B, C.** Posaconazole protruding from the tunnel entrance is shown in the chain B (**B**) and chain D (**C**). Protein backbone is depicted by yellow ribbon, protein surface is represented by black mesh, residues surrounding tunnel entrance (cyan) are in stick mode. Arrows point at the residues corresponding mutation hot spots in posaconazole resistant isolates of *A. fumigatus* and *C. albicans*. Posaconazole is shown by spheres with the carbon atoms white, oxygen red, nitrogen blue, fluorine light green. Heme in the background is orange. K50 side chain is not defined in the electron density and therefore modeled as alanine. Images were prepared using MAESTRO [63].

The CYP51_Tc_ structures revealed a 42 residue-long hydrophobic tunnel connecting the chamber adjacent to the heme with the protein surface (Fig. 6B). Residues constituting the tunnel in addition to those interacting with fluconazole are labeled with green triangles in Fig. 7. The mouth of the channel is surrounded by residues I45, I46, G49, K50, I209, P210, H458, and M460, which may delineate the substrate/inhibitor entry site in eukaryotic CYP51 (Fig. 8). This entry mode would be in contrast to that in CYP51_Mt_, where it most likely occurs through the open BC-loop. The tunnel-forming residues are invariant between CYP51_Tc_ and CYP51_Tb_ with the exception of four conservative substitutions at positions 46, 105, 215 and 359. The residue at position 105 (highlighted cyan in Fig. 7) is known to dominate substrate specificity with respect to the methylation status of the C-4 atom in CYP51 sterol substrates. I105 in *T. cruzi* allows efficient conversion of C4-dimethylated 24-methylenedihydrolanosterol while the bulkier F105 in *T. brucei* favors C4-monomethylated norlanosterol [19], [21]. Phenylalanine in CYP51_Tb_ protrudes further into the active site than isoleucine in CYP51_Tc_ (Fig. 6A), potentially resulting in interference with the 4β-methyl group of the sterol substrate. However, F105 does not interfere with either posaconazole or fluconazole binding.

Comparison of the residues constituting the tunnel in CYP51_Tc_ with the human counterpart, CYP51_h_, indicates that two residues, H236 and H489 (numbered according to the human sequence and highlighted yellow in Fig. 7), protrude into the tunnel near the opening, reducing both its size and hydrophobicity. As they are present exclusively in mammalian orthologues [41], H236 and H489 may partly account for the selectivity of azole drugs toward pathogenic fungi and protozoa. In accord with this hypothesis, proline corresponding to H236 in pathogenic fungi is among hot spots that confer resistance to posaconazole in *Aspergillus fumigatus* (P216) [42] and *Candida albicans* (P230) [43].

### Posaconazole binding

**Figure 9:**
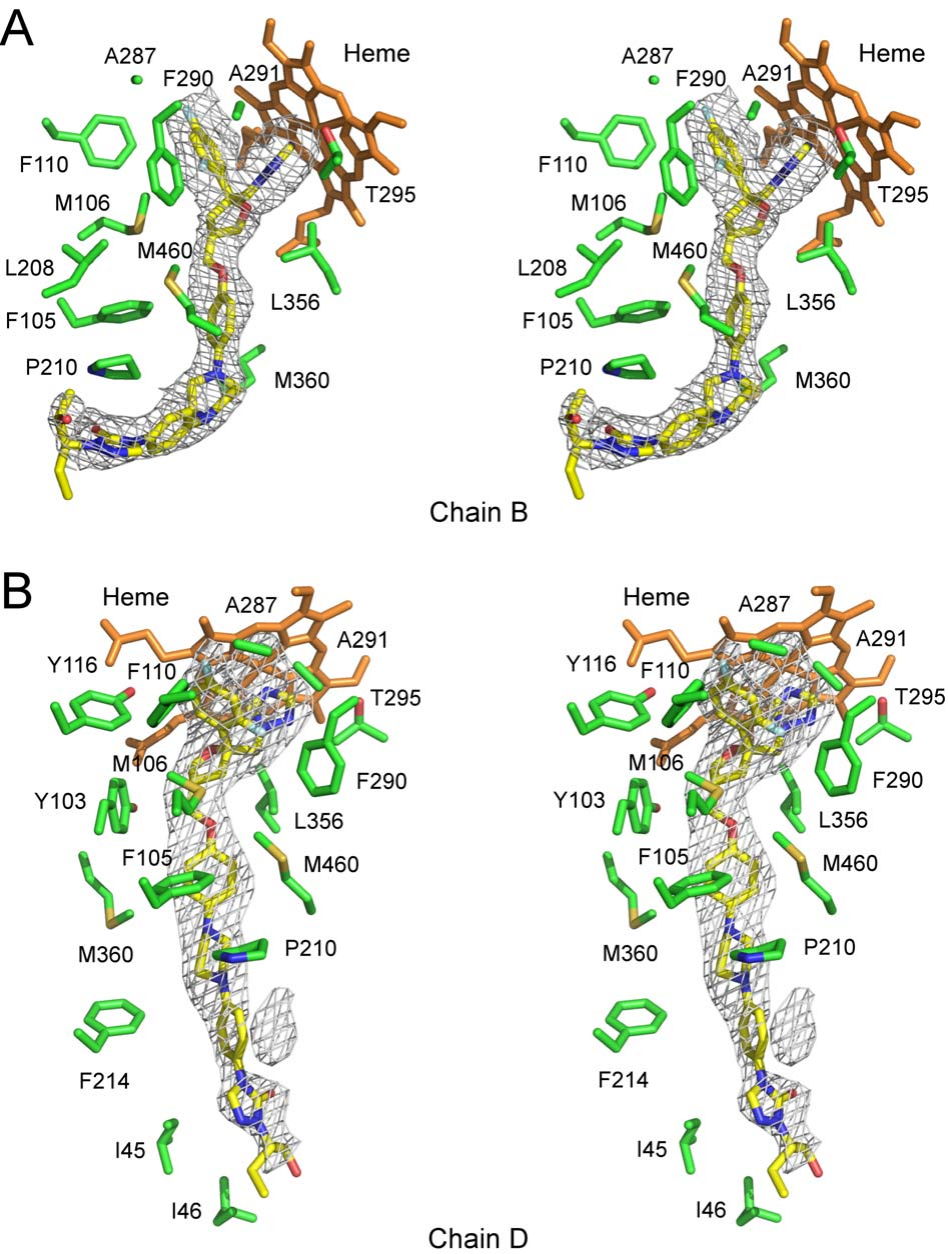
Posaconazole binding. Stereoscopic view of posaconazole in chain B (**A**) and in chain D (**B**) surrounded by the side chains within 4 Å. Image in **B** is rotated ∼90° to the right along the vertical axis in the plane of drawing. To avoid image cluttering, four residues from this range, Y103, Y116, A211 and M360, were excluded from the view in **A**. Posaconazole carbons are highlighted in yellow, amino acid residues are in green. Otherwise color scheme is as in **Fig. 8**.

The hydrophobic tunnel in CYP51_Tb_ accommodates the antifungal drug posaconazole in either extended or bent conformations. The 2.6 Å structure of the CYP51_Tb_-posaconazole complex revealed four protein monomers in an asymmetric unit with posaconazole coordinating to the heme iron in a manner similar to that of fluconazole with the fluorinated edge of the 2,4-difluorophenyl ring facing away from the heme macrocycle, and the long substituent tail extending into the hydrophobic tunnel. Electron density is well defined for the Fe-coordinating head of the posaconazole molecule in all four monomers but somewhat fades out toward its long tail (Fig. 8A). Thus, the terminal 2-hydroxypentan group is defined in none of four monomers. Three from the four monomers (chains A, B, and C) accommodate posaconazole in bent conformation while in the monomer D posaconazole is in extended conformation. Conformational variability of posaconazole is enabled by the interconversion of the piperazine six-membered ring between the chair and twisted boat conformations. The latter serves to accommodate the bend. Electron density is best defined in monomer B, where the terminal phenyl-2-hydroxypentan-triazolone group of posaconazole lies within 6 Å of protein residues I209-P210-A211 and V213-F214 which are invariant between CYP51_Tc_ and CYP51_Tb_. P210, the mutation hot spot in fungi, is situated right in the bend of the posaconazole molecule (Fig. 9A). In the extended conformation in monomer D, the phenyl-2-hydroxypentan-triazolone group swings toward residues I45-I46 (Fig. 9B). Remarkably, points of posaconazole contact in the tunnel mouth are among mutation hot spots in azole resistant isolates of pathogenic fungi *A. fumigatus* [42], [44]–[48] and *C. albicans* [43], [49] (Fig. 8B and C).The scattered F_o_-F_c_ electron density map in the monomers A and D (Fig. 8A) suggests possible interconversion of the posaconazole conformers in dynamic equilibrium, meaning that the phenyl-2-hydroxypentan-triazolone group dangles in space within the tunnel mouth (Fig. 3D). Given the high sequence and structural similarities between CYP51_Tc_ and CYP51_Tb_, similar dynamics would be expected in the CYP51_Tc_-posaconazole complex.

### Docking studies

**Figure 10:**
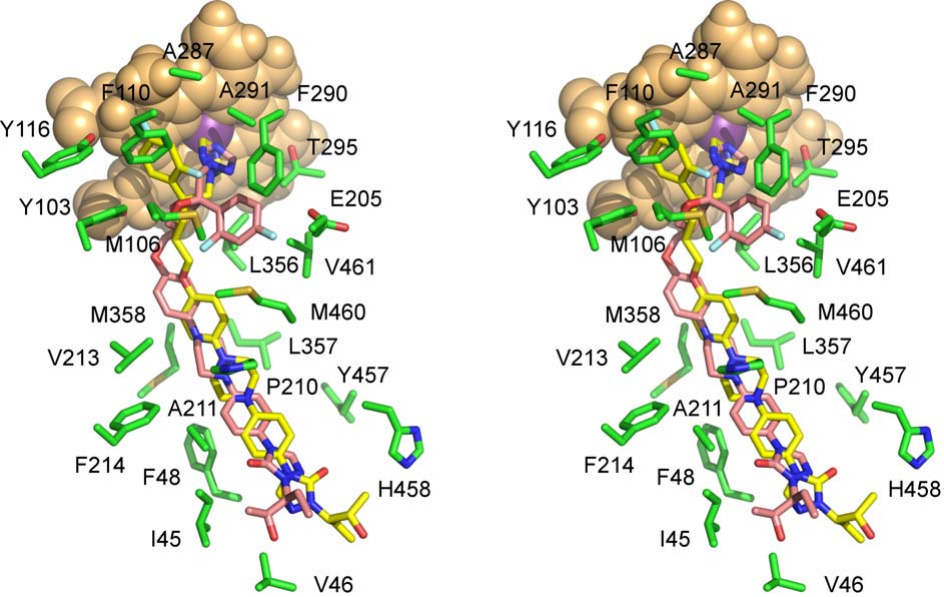
Posaconazole docking. Stereoscopic view of posaconazole docked in the active site CYP51_Tc_ in **pose 1** (yellow) and **pose 2** (pink). Residues within 4 Å of posaconazole (green) are shown. Binding ambiguity of the long posaconazole tail is likely due to the hydrophobic/aromatic stacking nature of the interactions in the substrate binding tunnel. Posaconazole and protein are shown by stick, heme by van der Waals spheres. Posaconazole carbon atoms are yellow or pink, oxygen red, nitrogen blue, sulfur yellow and fluorine pale cyan; heme is orange, For clarity, residues L208 and M360 are excluded from the view.

The x-ray structures of the CYP51 therapeutic targets determined in this work are intended for use in rational drug design. We also apply computational methods to explore binding modes of known chemical structures as well as to generate new scaffolds based on the configuration of the CYP51 binding sites. Considering the differential geometries of the host and pathogen binding sites, we aim to develop a pool of highly selective molecules with no cross-reactivity to human CYP51. As a first step, we docked posaconazole into the CYP51_Tc_ active site and compared the docking poses with the experimental structure of CYP51_Tb_-posaconazole complex. Two poses with similar docking scores were identified for posaconazole by GLIDE [39], differing primarily in the orientation of the 2,4-difluorophenyl ring (Fig. 10). Interestingly, the long posaconazole tail docks in a mode more similar to the CYP51_Tb_-posaconazole complex defined in this work rather than that in the recently deposited *T. cruzi* structure (PDB ID Code: 3K1O). Given that the protein-posaconazole interactions in the tunnel are of hydrophobic/aromatic stacking nature (Fig. 9), this ambiguity is not surprising.

Another source of docking ambiguity arises from the binding predicted for the 2,4-difluorophenyl substituent. In the better scoring **pose** **1** (highlighted yellow in Fig. 10), the 2,4-difluorophenyl ring binds in the experimentally observed orientation 1. In the slightly lower scoring **pose 2** (highlighted pink), the 2,4-difluorophenyl ring is bound in a different pocket formed by the residues M106, E205, L208, F290, T295, L358 and M460, suggesting an additional cavity in the CYP51 active site suitable for drug targeting. This pose is achieved via flipping of the central furan ring to which all the substituents are attached. Thus, in addition to the experimentally observed binding ambiguity of the long substituent tail, conformational ambiguity of the difluorophenyl ring is predicted by the docking calculations and perhaps will be observed in future structures of CYP51 in complex with inhibitors similar to posaconazole.

### Implications for drug resistance

The rapid development of azole resistance in *T. cruzi* observed *in vitro* suggests that the same may occur in patients [50]. Although no data are available on the development of posaconazole resistance in Chagas Disease patients, studies conducted on fungal infections indicate that posaconazole resistance occurs mainly by a mechanism involving mutation of the *cyp51* gene [42], [51], [52]. Posaconazole appears to be less susceptible to the efflux pumps that confer resistance to some other azoles [43], [51], [53]. Mapping mutations in *cyp51* genes in clinical posaconazole resistant isolates on the CYP51 structure, points to the tunnel entrance as a mutation hot spot. Mutations of G54, P216 and M220 in clinical isolates of *A. fumigatus* [42], [44]–[48] (corresponding to G49, P210 and F214, respectively, in CYP51_Tc_ and CYP51_Tb_) and of A61 [49] and P230 [43] in clinical isolates of *C. albicans* (I45 and P210, respectively, in CYP51_Tc_ and CYP51_Tb_) map directly to the tunnel mouth (Fig. 8B and C). Mutations of G54 in *A. fumigatus* to arginine or tryptophan associate with moderate and high levels of resistance, respectively, and confer cross-resistance between itraconazole and posaconazole [44]. Mutations of M220 confer cross-resistance to all azole drugs tested, including itraconazole, voriconazole, ravuconazole and posaconazole [54], [55] and therefore may interfere with the entry of the drugs. In accord with this assumption, posaconazole is reported to induce resistance to all azole drugs in *Candida parapsilosis in vitro* [51]. The alarming perspective emerging from antifungal therapy efforts must be taken into consideration when designing anti-Chagasic drugs targeting CYP51_Tc_. Thus, the terminal phenyl-2-hydroxypentan-triazolone group in posaconazole may play an important role in pharmacokinetics rather than in the interactions with the target, and yet these interactions seem to induce resistance which otherwise could probably be avoided.

In summary, the x-ray structures of *Trypanosoma* CYP51 enzymes reported here open new opportunities for rationally designed inhibitors against therapeutic targets in important human pathogens. The structures provide templates for developing CYP51 inhibitors with improved efficacy and resistance properties that are structurally and synthetically simpler than posaconazole. By utilizing the differential geometries between host and pathogen CYP51 binding sites, it maybe possible to create new drugs with minimized toxicity and host-pathogen cross-reactivity. In addition, the posaconazole binding mode offers insights into the development of drug resistance in pathogenic fungi, implying that an analogous mechanism may be implicated in protozoan pathogens. The reported structures also provide a good template for drug design targeting *Leishmania* CYP51. However, drug development must take into account the properties and accessibility of the compartment where these parasites reside. Unlike *T. cruzi*, *Leishmania* amastigotes replicate in the acidic environment (pH ∼5) of the phagolysosomal vacuoles in macrophage cells [56], [57], imposing different requirements on the physicochemical properties of CYP51 inhibitors targeting leishmaniasis.
